# 6-(1-Methyl­ethyl)-12-phenyl-5,6,7,12-tetra­hydro­dibenz[*c*,*f*][1,5]aza­silocine

**DOI:** 10.1107/S1600536809050909

**Published:** 2009-12-04

**Authors:** Kei Goto, Akihiro Fukushima, Takayuki Kawashima

**Affiliations:** a Interactive Research Center of Science, Graduate School of Science and Engineering, Tokyo Instiute of Technology, Ookayama, Meguro-ku, Tokyo, 152-8551, Japan; b Department of Chemistry, Graduate School of Science, The University of Tokyo, Hongo, Bunkyo-ku, Tokyo 113-0033, Japan

## Abstract

The title compound, C_23_H_25_NSi, has an eight-membered silicon-containing heterocyclic ring with an intra­molecular N⋯Si close contact, the transannular distance of which is 2.6294 (18) Å. The resulting geometry about the Si atom is distorted trigonal-bypyramidal, with the N and H atoms occupying apical sites. The dihedral angle between the aromatic rings fused to the eight-membered ring is 63.27 (7)°.

## Related literature

For highly coordinated organosilanes, see: Brellère *et al.* (1986 ▶); Carré *et al.* (1997 ▶); Paton *et al.* (1977 ▶); Woning & Verkade (1991 ▶); Yoshida *et al.* (2006 ▶). For a related structure, see: Saruhashi *et al.* (2001 ▶).
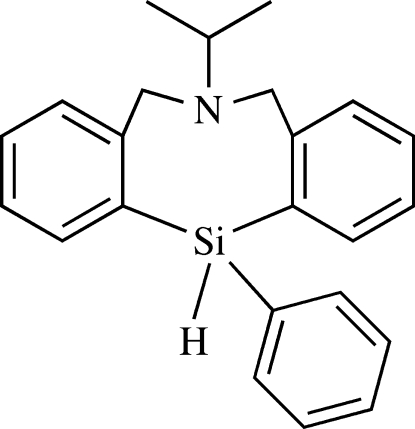


## Experimental

### 

#### Crystal data

C_23_H_25_NSi
*M* *_r_* = 343.53
Monoclinic, 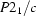

*a* = 9.756 (7) Å
*b* = 10.269 (7) Å
*c* = 18.912 (12) Å
β = 92.745 (3)°
*V* = 1893 (2) Å^3^
*Z* = 4
Mo *K*α radiation
μ = 0.13 mm^−1^
*T* = 120 K
0.20 × 0.20 × 0.10 mm

#### Data collection

Rigaku Mercury CCD diffractometer
Absorption correction: multi-scan (*REQAB*; Jacobson, 1998 ▶) *T*_min_ = 0.975, *T*_max_ = 0.987
11962 measured reflections
3278 independent reflections
2798 reflections with *I* > 2σ(*I*)
*R*_int_ = 0.029

#### Refinement

*R*[*F* ^2^ > 2σ(*F* ^2^)] = 0.041
*wR*(*F* ^2^) = 0.100
*S* = 1.08
3278 reflections
232 parameters
H atoms treated by a mixture of independent and constrained refinement
Δρ_max_ = 0.30 e Å^−3^
Δρ_min_ = −0.30 e Å^−3^

### 

Data collection: *CrystalClear* (Rigaku, 2004 ▶); cell refinement: *CrystalClear* data reduction: *CrystalClear*; program(s) used to solve structure: *SHELXS97* (Sheldrick, 2008 ▶); program(s) used to refine structure: *SHELXL97* (Sheldrick, 2008 ▶); molecular graphics: *ORTEPIII* (Burnett & Johnson, 1996 ▶); software used to prepare material for publication: *yadokari-XG* (Wakita, 2005 ▶).

## Supplementary Material

Crystal structure: contains datablocks I, global. DOI: 10.1107/S1600536809050909/is2498sup1.cif

Structure factors: contains datablocks I. DOI: 10.1107/S1600536809050909/is2498Isup2.hkl

Additional supplementary materials:  crystallographic information; 3D view; checkCIF report

## Figures and Tables

**Fig. 1.**
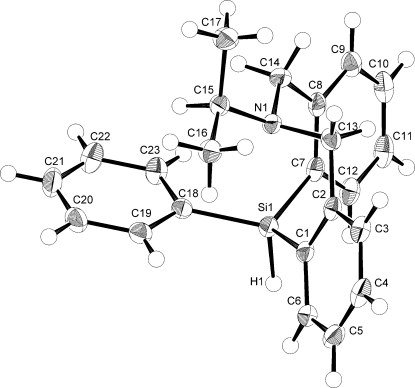
The molecular structure of (I) with 50% probability displacement ellipsoids (arbitrary spheres for H atoms).

**Fig. 2.**
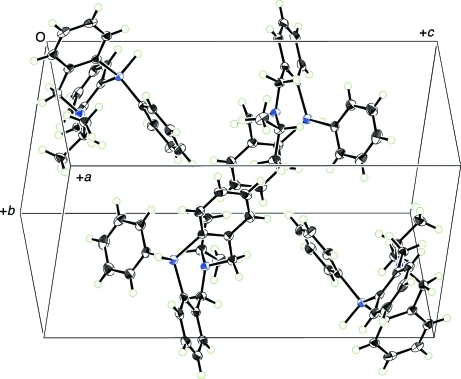
Packing diagram.

## supplementary crystallographic information

### Comment

Highly coordinated hydrosilanes have been of great interest for their unique structures and reactivities. It has been known that, in highly coordinated monohydrosilanes, the Si—H bond has high affinity for equatorial position (Brellère *et al.*, 1986) and there are only a few examples with the Si—H bond at the apical position (Woning & Verkade, 1991). A dibenz[*c*,*f*][1,5]azasilocine framework has been utilized for the synthesis of various highly coordinated silicon compounds (Paton *et al.*, 1977; Carré *et al.*, 1997; Yoshida *et al.*, 2006). Recently, we reported the synthesis and structural characterization of a pentacoordinated monohydrosilane bearing this molecular framework with the apical Si—H bond (Saruhashi *et al.*, 2001). As a further investigation of this work, the crystal structure of the title new hydrosilane is reported.

The title compound was synthesized by the reaction of *N*,*N*-bis(2-bromobenzyl)isopropylamine (Carré *et al.*, 1997) with *n*-butyllithium followed by treatment with phenylsilane. The molecular structure of the title compound is shown in Fig. 1. It was found that the geometry around the silicon atom is that of a distorted trigonal bypyramid with the sum of the equatorial C—Si—C bond angles of 346.3°. The SiH hydrogen atom occupies the apical site in spite of its lower apicophilicity than that of a phenyl group, which is similar to the related *N*-butyl compound we previously reported (Saruhashi *et al.*, 2001). The Si···N transannular distance is 2.6294 (18) Å, which is slightly longer than that of the *N*-butyl derivative [2.516 (2) Å] probably because of the steric repulsion between the isopropyl group and the phenyl ring.

### Experimental

A solution of *n*-butyllithium in hexane (1.6 *M*; 4.2 ml, 6.7 mmol) was added dropwise to a solution of *N*,*N*-bis(2-bromobenzyl)isopropylamine (1.25 g, 3.16 mmol) in ether (3 ml) at 233 K. The solution was stirred at the same temperature for 30 min and then allowed to warm to room temperature. After stirring for additional 2 h, the solution was cooled to 233 K, and a solution of phenylsilane (345 mg, 3.19 mmol) in ether (2 ml) was added dropwise. The mixture was allowed to warm to room temperature, and stirred overnight. After addition of water, the mixture was extracted with ether, and the organic layer was dried over anhydrous magnesium sulfate. After filtration and removal of the solvent, the residue was purified by gel permeation liquid chromatography (eluting with chloroform) and then recrystallization from hexane to give the title compound (101 mg, 0.295 mmol, 9.3%) as colorless crystals. Physical data: m.p. 354.1–355.8 K (decomposition); ^1^NMR (400 MHz, CDCl_3_, 300 K): δ 0.76 (br, 6H), 2.53 (br, 1H), 3.78 (s, 4H), 5.53 (brs, 1H), 7.12–7.32 (m, 9H), 7.51 (br, 2H), 7.72 (br, 2H). Anal. Calcd for C_23_H_25_NSi: C 80.41, H 7.34, N 4.08%. Found: C 80.19, H 7.48, N, 3.94%.

### Refinement

The H atom of the SiH group was found in a difference Fourier map and refined isotropically, while the C-bound H atoms were treated as riding, with C—H = 0.95–0.99 Å, and with *U*_iso_(H) = 1.2 (1.5 for methyl groups) times *U*_eq_(C). The methyl groups were allowed to rotate freely about the C-C bond.

### Crystal data

**Table:**

C_23_H_25_NSi	*Z* = 4
*M**_r_* = 343.53	*F*(000) = 736
Monoclinic, *P*2_1_/*c*	*D*_x_ = 1.206 Mg m^−^^3^
Hall symbol: -P 2ybc	Mo *K*α radiation, λ = 0.71070 Å
*a* = 9.756 (7) Å	θ = 3.0–25.0°
*b* = 10.269 (7) Å	µ = 0.13 mm^−^^1^
*c* = 18.912 (12) Å	*T* = 120 K
β = 92.745 (3)°	Block, colourless
*V* = 1893 (2) Å^3^	0.20 × 0.20 × 0.10 mm

### Data collection

**Table:**

Rigaku Mercury CCD diffractometer	3278 independent reflections
Radiation source: fine-focus sealed tube	2798 reflections with *I* > 2σ(*I*)
graphite	*R*_int_ = 0.029
ω scans	θ_max_ = 25.0°, θ_min_ = 3.0°
Absorption correction: multi-scan (*REQAB*; Jacobson, 1998)	*h* = −11→11
*T*_min_ = 0.975, *T*_max_ = 0.987	*k* = −12→9
11962 measured reflections	*l* = −21→21

### Refinement

**Table:**

Refinement on *F*^2^	Primary atom site location: structure-invariant direct methods
Least-squares matrix: full	Secondary atom site location: difference Fourier map
*R*[*F*^2^ > 2σ(*F*^2^)] = 0.041	Hydrogen site location: inferred from neighbouring sites
*wR*(*F*^2^) = 0.100	H atoms treated by a mixture of independent and constrained refinement
*S* = 1.08	*w* = 1/[σ^2^(*F*_o_^2^) + (0.0402*P*)^2^ + 1.0221*P*] where *P* = (*F*_o_^2^ + 2*F*_c_^2^)/3
3278 reflections	(Δ/σ)_max_ < 0.001
232 parameters	Δρ_max_ = 0.30 e Å^−^^3^
0 restraints	Δρ_min_ = −0.30 e Å^−^^3^

### Special details

**Table:**

Geometry. All e.s.d.'s (except the e.s.d. in the dihedral angle between two l.s. planes) are estimated using the full covariance matrix. The cell e.s.d.'s are taken into account individually in the estimation of e.s.d.'s in distances, angles and torsion angles; correlations between e.s.d.'s in cell parameters are only used when they are defined by crystal symmetry. An approximate (isotropic) treatment of cell e.s.d.'s is used for estimating e.s.d.'s involving l.s. planes.
Refinement. Refinement of *F*^2^ against ALL reflections. The weighted *R*-factor *wR* and goodness of fit *S* are based on *F*^2^, conventional *R*-factors *R* are based on *F*, with *F* set to zero for negative *F*^2^. The threshold expression of *F*^2^ > σ(*F*^2^) is used only for calculating *R*-factors(gt) *etc*. and is not relevant to the choice of reflections for refinement. *R*-factors based on *F*^2^ are statistically about twice as large as those based on *F*, and *R*- factors based on ALL data will be even larger.

### Fractional atomic coordinates and isotropic or equivalent isotropic displacement parameters (Å^2^)

**Table:**

	*x*	*y*	*z*	*U*_iso_*/*U*_eq_	
Si1	0.10808 (5)	0.12541 (5)	0.18691 (3)	0.02030 (15)	
H1	0.0293 (18)	0.0512 (18)	0.2351 (9)	0.023 (5)*	
C1	0.14424 (17)	−0.00037 (17)	0.11833 (9)	0.0204 (4)	
C2	0.17207 (17)	0.03310 (17)	0.04842 (9)	0.0210 (4)	
C3	0.19436 (18)	−0.06347 (19)	−0.00083 (10)	0.0260 (4)	
H2	0.2108	−0.0400	−0.0483	0.031*	
C4	0.19306 (18)	−0.19363 (19)	0.01801 (11)	0.0286 (4)	
H3	0.2105	−0.2589	−0.0160	0.034*	
C5	0.16617 (19)	−0.22838 (18)	0.08669 (11)	0.0297 (5)	
H4	0.1659	−0.3175	0.1001	0.036*	
C6	0.13964 (18)	−0.13231 (17)	0.13580 (10)	0.0239 (4)	
H5	0.1179	−0.1569	0.1824	0.029*	
C7	−0.01585 (18)	0.25743 (17)	0.15815 (9)	0.0213 (4)	
C8	0.02098 (18)	0.36529 (17)	0.11797 (9)	0.0216 (4)	
C9	−0.07488 (19)	0.46221 (18)	0.10020 (10)	0.0263 (4)	
H6	−0.0488	0.5345	0.0725	0.032*	
C10	−0.20760 (19)	0.4539 (2)	0.12252 (10)	0.0305 (5)	
H7	−0.2716	0.5213	0.1113	0.037*	
C11	−0.24688 (19)	0.3469 (2)	0.16127 (10)	0.0311 (5)	
H8	−0.3385	0.3398	0.1758	0.037*	
C12	−0.15189 (18)	0.25012 (19)	0.17883 (9)	0.0256 (4)	
H9	−0.1797	0.1771	0.2055	0.031*	
C13	0.17620 (17)	0.17561 (17)	0.02855 (9)	0.0223 (4)	
H10	0.2334	0.1872	−0.0128	0.027*	
H11	0.0823	0.2062	0.0153	0.027*	
C14	0.16617 (18)	0.37943 (17)	0.09530 (10)	0.0230 (4)	
H12	0.1655	0.4257	0.0494	0.028*	
H13	0.2192	0.4327	0.1306	0.028*	
N1	0.23330 (14)	0.25313 (14)	0.08823 (7)	0.0196 (3)	
C15	0.38542 (17)	0.26790 (18)	0.08917 (10)	0.0231 (4)	
H14	0.4150	0.3025	0.1369	0.028*	
C16	0.45915 (18)	0.13896 (18)	0.08041 (10)	0.0267 (4)	
H15	0.4229	0.0745	0.1128	0.040*	
H16	0.5576	0.1508	0.0912	0.040*	
H17	0.4445	0.1084	0.0315	0.040*	
C17	0.4350 (2)	0.3648 (2)	0.03466 (11)	0.0331 (5)	
H18	0.4060	0.3354	−0.0130	0.050*	
H19	0.5353	0.3705	0.0387	0.050*	
H20	0.3954	0.4507	0.0434	0.050*	
C18	0.25657 (18)	0.18885 (17)	0.24348 (9)	0.0210 (4)	
C19	0.37831 (18)	0.11897 (18)	0.25521 (9)	0.0253 (4)	
H21	0.3880	0.0367	0.2330	0.030*	
C20	0.48570 (19)	0.1670 (2)	0.29869 (10)	0.0317 (5)	
H22	0.5678	0.1178	0.3056	0.038*	
C21	0.4733 (2)	0.2858 (2)	0.33186 (11)	0.0361 (5)	
H23	0.5466	0.3186	0.3616	0.043*	
C22	0.3540 (2)	0.3568 (2)	0.32163 (11)	0.0356 (5)	
H24	0.3448	0.4386	0.3444	0.043*	
C23	0.24691 (19)	0.30841 (19)	0.27785 (10)	0.0281 (4)	
H25	0.1652	0.3581	0.2712	0.034*	

### Atomic displacement parameters (Å^2^)

**Table:**

	*U*^11^	*U*^22^	*U*^33^	*U*^12^	*U*^13^	*U*^23^
Si1	0.0192 (3)	0.0210 (3)	0.0206 (3)	−0.0018 (2)	−0.00047 (19)	−0.0006 (2)
C1	0.0149 (8)	0.0215 (9)	0.0243 (10)	−0.0014 (7)	−0.0028 (7)	−0.0001 (8)
C2	0.0130 (8)	0.0251 (10)	0.0246 (10)	−0.0005 (7)	−0.0015 (7)	−0.0029 (8)
C3	0.0195 (9)	0.0328 (11)	0.0256 (10)	−0.0031 (8)	−0.0001 (7)	−0.0060 (8)
C4	0.0201 (9)	0.0293 (10)	0.0361 (12)	−0.0003 (8)	−0.0025 (8)	−0.0141 (9)
C5	0.0238 (10)	0.0184 (9)	0.0460 (13)	−0.0011 (8)	−0.0079 (9)	−0.0037 (9)
C6	0.0212 (9)	0.0233 (10)	0.0268 (10)	−0.0031 (7)	−0.0044 (7)	0.0023 (8)
C7	0.0201 (9)	0.0248 (9)	0.0189 (9)	−0.0010 (7)	−0.0019 (7)	−0.0079 (8)
C8	0.0215 (9)	0.0220 (9)	0.0207 (9)	0.0015 (7)	−0.0046 (7)	−0.0063 (8)
C9	0.0290 (10)	0.0235 (10)	0.0256 (10)	0.0036 (8)	−0.0055 (8)	−0.0070 (8)
C10	0.0255 (10)	0.0353 (11)	0.0297 (11)	0.0100 (9)	−0.0081 (8)	−0.0127 (9)
C11	0.0179 (9)	0.0450 (12)	0.0300 (11)	0.0035 (9)	−0.0020 (8)	−0.0149 (10)
C12	0.0238 (10)	0.0327 (11)	0.0203 (10)	−0.0022 (8)	0.0007 (7)	−0.0094 (8)
C13	0.0188 (9)	0.0274 (10)	0.0206 (10)	−0.0002 (7)	0.0007 (7)	0.0020 (8)
C14	0.0234 (9)	0.0183 (9)	0.0272 (10)	−0.0002 (7)	−0.0011 (7)	0.0025 (8)
N1	0.0165 (7)	0.0190 (8)	0.0232 (8)	−0.0006 (6)	−0.0006 (6)	0.0012 (6)
C15	0.0162 (9)	0.0270 (10)	0.0261 (10)	−0.0027 (7)	−0.0007 (7)	0.0053 (8)
C16	0.0172 (9)	0.0296 (10)	0.0334 (11)	0.0003 (8)	0.0029 (8)	0.0043 (9)
C17	0.0226 (10)	0.0357 (11)	0.0410 (12)	−0.0047 (9)	0.0020 (8)	0.0129 (10)
C18	0.0220 (9)	0.0243 (9)	0.0168 (9)	−0.0031 (7)	0.0018 (7)	0.0027 (8)
C19	0.0262 (10)	0.0259 (10)	0.0238 (10)	−0.0007 (8)	0.0015 (8)	0.0040 (8)
C20	0.0211 (10)	0.0394 (12)	0.0341 (12)	−0.0002 (8)	−0.0043 (8)	0.0106 (10)
C21	0.0276 (11)	0.0470 (13)	0.0328 (12)	−0.0103 (10)	−0.0081 (9)	0.0000 (10)
C22	0.0334 (11)	0.0378 (12)	0.0352 (12)	−0.0057 (9)	−0.0035 (9)	−0.0114 (10)
C23	0.0233 (10)	0.0324 (11)	0.0285 (11)	0.0012 (8)	−0.0007 (8)	−0.0047 (9)

### Geometric parameters (Å, °)

**Table:**

Si1—C1	1.876 (2)	C13—H10	0.9900
Si1—C18	1.876 (2)	C13—H11	0.9900
Si1—C7	1.880 (2)	C14—N1	1.462 (2)
Si1—H1	1.438 (18)	C14—H12	0.9900
C1—C6	1.396 (3)	C14—H13	0.9900
C1—C2	1.405 (3)	N1—C15	1.491 (2)
C2—C3	1.385 (3)	C15—C16	1.520 (3)
C2—C13	1.512 (3)	C15—C17	1.528 (3)
C3—C4	1.384 (3)	C15—H14	1.0000
C3—H2	0.9500	C16—H15	0.9800
C4—C5	1.384 (3)	C16—H16	0.9800
C4—H3	0.9500	C16—H17	0.9800
C5—C6	1.388 (3)	C17—H18	0.9800
C5—H4	0.9500	C17—H19	0.9800
C6—H5	0.9500	C17—H20	0.9800
C7—C8	1.400 (3)	C18—C23	1.394 (3)
C7—C12	1.403 (3)	C18—C19	1.396 (3)
C8—C9	1.396 (3)	C19—C20	1.391 (3)
C8—C14	1.506 (3)	C19—H21	0.9500
C9—C10	1.384 (3)	C20—C21	1.380 (3)
C9—H6	0.9500	C20—H22	0.9500
C10—C11	1.385 (3)	C21—C22	1.380 (3)
C10—H7	0.9500	C21—H23	0.9500
C11—C12	1.388 (3)	C22—C23	1.393 (3)
C11—H8	0.9500	C22—H24	0.9500
C12—H9	0.9500	C23—H25	0.9500
C13—N1	1.469 (2)		
			
Si1···N1	2.6294 (18)		
			
C1—Si1—C18	117.89 (8)	C2—C13—H11	109.6
C1—Si1—C7	115.86 (8)	H10—C13—H11	108.1
C18—Si1—C7	112.52 (9)	N1—C14—C8	111.83 (14)
C1—Si1—N1	75.14 (8)	N1—C14—H12	109.2
C18—Si1—N1	81.91 (8)	C8—C14—H12	109.2
C7—Si1—N1	75.47 (8)	N1—C14—H13	109.2
C1—Si1—H1	101.4 (7)	C8—C14—H13	109.2
C18—Si1—H1	104.2 (7)	H12—C14—H13	107.9
C7—Si1—H1	102.1 (7)	C14—N1—C13	113.33 (14)
N1—Si1—H1	173.9 (7)	C14—N1—C15	111.02 (14)
C6—C1—C2	118.03 (16)	C13—N1—C15	113.88 (14)
C6—C1—Si1	119.71 (14)	C14—N1—Si1	98.76 (11)
C2—C1—Si1	122.24 (14)	C13—N1—Si1	95.92 (11)
C3—C2—C1	120.09 (17)	C15—N1—Si1	122.57 (10)
C3—C2—C13	121.27 (17)	N1—C15—C16	112.71 (15)
C1—C2—C13	118.63 (15)	N1—C15—C17	113.89 (14)
C4—C3—C2	120.99 (18)	C16—C15—C17	109.11 (16)
C4—C3—H2	119.5	N1—C15—H14	106.9
C2—C3—H2	119.5	C16—C15—H14	106.9
C3—C4—C5	119.69 (18)	C17—C15—H14	106.9
C3—C4—H3	120.2	C15—C16—H15	109.5
C5—C4—H3	120.2	C15—C16—H16	109.5
C4—C5—C6	119.64 (18)	H15—C16—H16	109.5
C4—C5—H4	120.2	C15—C16—H17	109.5
C6—C5—H4	120.2	H15—C16—H17	109.5
C5—C6—C1	121.51 (18)	H16—C16—H17	109.5
C5—C6—H5	119.2	C15—C17—H18	109.5
C1—C6—H5	119.2	C15—C17—H19	109.5
C8—C7—C12	117.67 (17)	H18—C17—H19	109.5
C8—C7—Si1	123.33 (14)	C15—C17—H20	109.5
C12—C7—Si1	118.98 (14)	H18—C17—H20	109.5
C9—C8—C7	120.56 (17)	H19—C17—H20	109.5
C9—C8—C14	119.41 (17)	C23—C18—C19	117.03 (16)
C7—C8—C14	120.01 (15)	C23—C18—Si1	120.26 (14)
C10—C9—C8	120.55 (19)	C19—C18—Si1	122.68 (14)
C10—C9—H6	119.7	C20—C19—C18	121.52 (18)
C8—C9—H6	119.7	C20—C19—H21	119.2
C9—C10—C11	119.83 (18)	C18—C19—H21	119.2
C9—C10—H7	120.1	C21—C20—C19	120.14 (18)
C11—C10—H7	120.1	C21—C20—H22	119.9
C10—C11—C12	119.74 (18)	C19—C20—H22	119.9
C10—C11—H8	120.1	C22—C21—C20	119.69 (18)
C12—C11—H8	120.1	C22—C21—H23	120.2
C11—C12—C7	121.62 (19)	C20—C21—H23	120.2
C11—C12—H9	119.2	C21—C22—C23	119.9 (2)
C7—C12—H9	119.2	C21—C22—H24	120.1
N1—C13—C2	110.32 (14)	C23—C22—H24	120.1
N1—C13—H10	109.6	C22—C23—C18	121.72 (18)
C2—C13—H10	109.6	C22—C23—H25	119.1
N1—C13—H11	109.6	C18—C23—H25	119.1
			
C18—Si1—C1—C6	90.22 (15)	C7—C8—C14—N1	27.9 (2)
C7—Si1—C1—C6	−132.20 (14)	C8—C14—N1—C13	68.82 (19)
N1—Si1—C1—C6	162.45 (15)	C8—C14—N1—C15	−161.54 (14)
C18—Si1—C1—C2	−91.80 (15)	C8—C14—N1—Si1	−31.55 (15)
C7—Si1—C1—C2	45.78 (17)	C2—C13—N1—C14	−142.27 (15)
N1—Si1—C1—C2	−19.57 (13)	C2—C13—N1—C15	89.55 (17)
C6—C1—C2—C3	−0.2 (2)	C2—C13—N1—Si1	−40.07 (14)
Si1—C1—C2—C3	−178.19 (13)	C1—Si1—N1—C14	148.32 (11)
C6—C1—C2—C13	179.50 (15)	C18—Si1—N1—C14	−89.90 (12)
Si1—C1—C2—C13	1.5 (2)	C7—Si1—N1—C14	25.98 (11)
C1—C2—C3—C4	−1.6 (3)	C1—Si1—N1—C13	33.57 (10)
C13—C2—C3—C4	178.74 (16)	C18—Si1—N1—C13	155.34 (11)
C2—C3—C4—C5	1.4 (3)	C7—Si1—N1—C13	−88.77 (11)
C3—C4—C5—C6	0.5 (3)	C1—Si1—N1—C15	−89.73 (13)
C4—C5—C6—C1	−2.4 (3)	C18—Si1—N1—C15	32.04 (13)
C2—C1—C6—C5	2.2 (3)	C7—Si1—N1—C15	147.92 (14)
Si1—C1—C6—C5	−179.79 (13)	C14—N1—C15—C16	−178.81 (15)
C1—Si1—C7—C8	−79.90 (16)	C13—N1—C15—C16	−49.5 (2)
C18—Si1—C7—C8	59.91 (17)	Si1—N1—C15—C16	65.15 (18)
N1—Si1—C7—C8	−14.73 (13)	C14—N1—C15—C17	−53.8 (2)
C1—Si1—C7—C12	101.44 (15)	C13—N1—C15—C17	75.5 (2)
C18—Si1—C7—C12	−118.76 (14)	Si1—N1—C15—C17	−169.85 (12)
N1—Si1—C7—C12	166.60 (15)	C1—Si1—C18—C23	158.83 (14)
C12—C7—C8—C9	0.7 (2)	C7—Si1—C18—C23	19.91 (17)
Si1—C7—C8—C9	−177.93 (13)	N1—Si1—C18—C23	90.44 (15)
C12—C7—C8—C14	178.98 (16)	C1—Si1—C18—C19	−23.30 (18)
Si1—C7—C8—C14	0.3 (2)	C7—Si1—C18—C19	−162.22 (14)
C7—C8—C9—C10	0.6 (3)	N1—Si1—C18—C19	−91.69 (15)
C14—C8—C9—C10	−177.69 (17)	C23—C18—C19—C20	−0.6 (3)
C8—C9—C10—C11	−1.7 (3)	Si1—C18—C19—C20	−178.53 (14)
C9—C10—C11—C12	1.4 (3)	C18—C19—C20—C21	0.4 (3)
C10—C11—C12—C7	−0.1 (3)	C19—C20—C21—C22	−0.1 (3)
C8—C7—C12—C11	−1.0 (3)	C20—C21—C22—C23	−0.1 (3)
Si1—C7—C12—C11	177.76 (14)	C21—C22—C23—C18	0.0 (3)
C3—C2—C13—N1	−145.26 (16)	C19—C18—C23—C22	0.4 (3)
C1—C2—C13—N1	35.1 (2)	Si1—C18—C23—C22	178.38 (15)
C9—C8—C14—N1	−153.86 (16)		
